# Supplementary material for: Sublethal systemic LPS in mice enables gut-luminal pathogens to bloom through oxygen species-mediated microbiota inhibition
Source: Nat Commun. 2025 Mar 20;16:2760. doi: 10.1038/s41467-025-57979-0 (PMC11926250; doi:10.1038/s41467-025-57979-0)
Supplement: Supplementary file 1 — Supplementary Information [file 41467_2025_57979_MOESM1_ESM.pdf]

Supplementary Information

Sublethal systemic LPS in mice enables gut-luminal pathogens to bloom through oxygen species-mediated microbiota inhibition

Sanne Kroon[1], Dejan Malcic[1], Lena Weidert[1,2], Lea Bircher[2], Leonardo Boldt[3,4,5], Philipp Christen[1], Patrick Kiefer[1], Anna Sintsova[1], Bidong D. Nguyen[1], Manja Barthel[1], Yves Steiger[1], Melanie Clerc[1], Mathias K.-M. Herzog[1], Carmen Chen[6], Ersin Gül[1], Benoit Guery[6], Emma Slack[2], Shinichi Sunagawa[1], Julia A. Vorholt[1], Lisa Maier[3,4,5], Christophe Lacroix[2], Annika Hausmann[1,2,7]*, Wolf-Dietrich Hardt[1]*

Affiliations

[1]Institute of Microbiology, Department of Biology, ETH Zürich, Zürich, Switzerland

[2]Institute of Food, Nutrition and Health, Department of Health Sciences and Technology, ETH Zürich, Zürich, Switzerland

[3]Interfaculty Institute of Microbiology and Infection Medicine Tübingen, University of Tübingen, Tübingen, Germany

[4]M3 Research Center for Malignome, Metabolome and Microbiome, University Hospital Tübingen, Tübingen, Germany

[5]Cluster of Excellence 'Controlling Microbes to Fight Infections', University of Tübingen, Tübingen, Germany

[6]Infectious Diseases Service, Lausanne University Hospital and University of Lausanne, Lausanne, Switzerland

[7]reNEW - Novo Nordisk Foundation Center for Stem Cell Medicine, University of Copenhagen, Copenhagen, Denmark

*For correspondence: annika.hausmann@hest.ethz.ch and hardt@micro.biol.ethz.ch

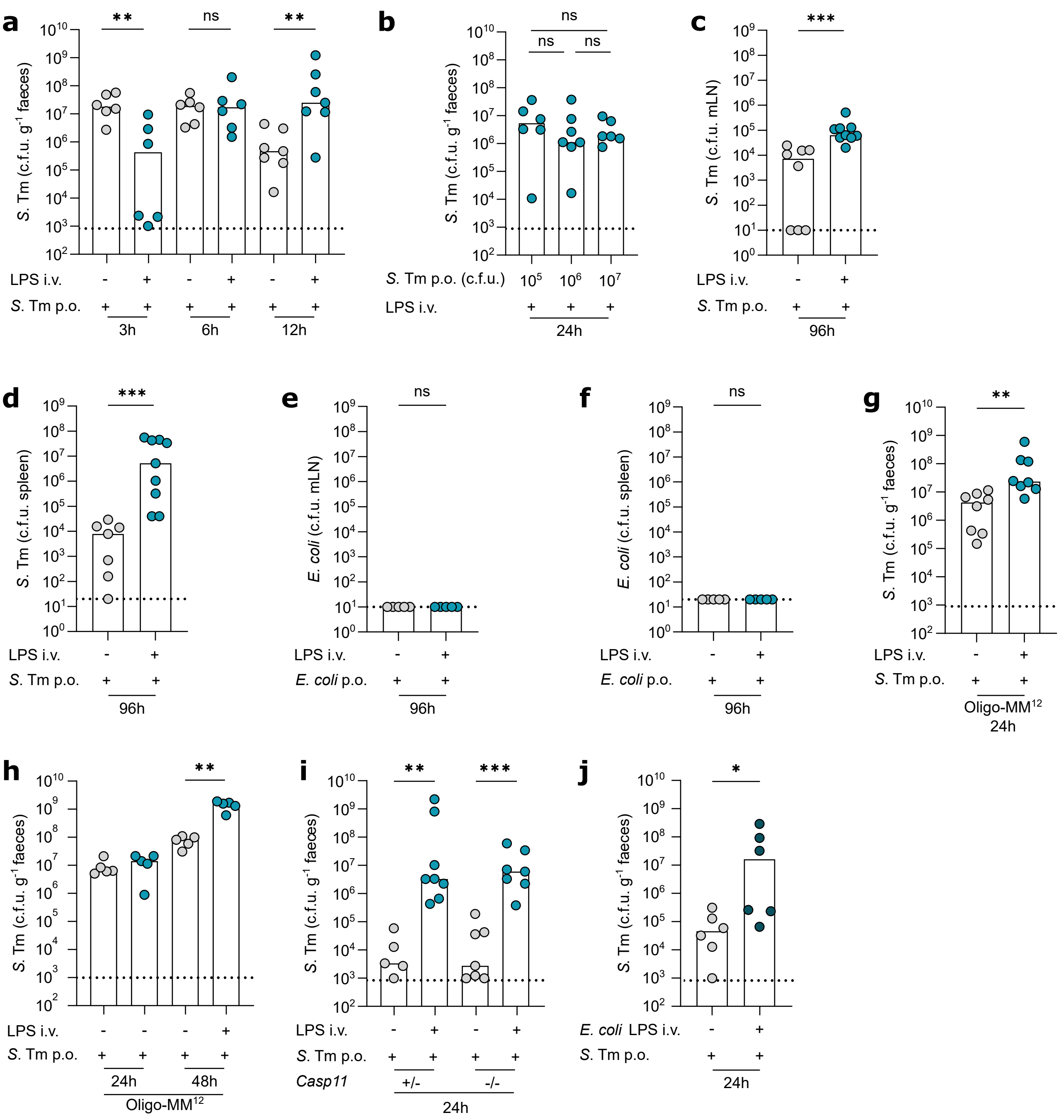

Supplementary Figure 1 Systemic LPS exposure promotes gut-luminal *S.* Tm to bloom. (a) Faecal *S.* Tm loads at 3, 6 or 12 h.p.i. in mice systemically exposed to PBS or LPS (minimum mice n=6, at least two independent replicates). (b) Faecal *S.* Tm loads at 24 h.p.i. in mice systemically exposed to LPS and orally infected with 10^5, 10^6 or 10^7 c.f.u. *S.* Tm (minimum mice n=6, at least two independent replicates). (c) Mesenteric lymph node (mLN) *S.* Tm loads at 24 h.p.i. in mice systemically exposed to PBS or LPS (minimum mice n=8, at least two independent replicates). (d) Spleen *S.* Tm loads at 24 h.p.i. in mice systemically exposed to PBS or LPS (minimum mice n=7, at least two independent replicates). (e) mLN *E. coli* loads at 24 h.p.i. in mice systemically exposed to PBS or LPS (mice n=5, at least two independent replicates). (f) Spleen *E. coli* loads at 24 h.p.i. in mice systemically exposed to PBS or LPS (mice n=5, at least two independent replicates). (g) Faecal *S.* Tm loads at 24 h.p.i. in Oligo-MM12 mice systemically exposed to PBS or LPS (mice n=8, at least two independent replicates). (h) Faecal *S.* Tm loads at 24 h and 48 h.p.i. in Oligo-MM12 mice orally infected with *S.* Tm and systemically exposed to PBS or LPS at 24 h.p.i. (mice n=5, at least two independent replicates). (i) Faecal *S.* Tm loads at 24 h.p.i. in *Caps11*$^{+/-}$ and *Caps11*$^{-/-}$ littermates systemically exposed to PBS or LPS (minimum mice n=5, at least two independent replicates). (j) Faecal S. Tm loads at 24 h.p.i. in mice systemically exposed to PBS or 5 µg E. coli LPS (mice n=6, at least two independent replicates). Bars indicate median values. Dotted lines indicate

conservative average limit of detection. *P* values were calculated using the two-sided Mann-Whitney U test (a,c-j) or two-sided Kruskal-Wallis test with Dunn's multiple comparisons adjustment (b). ns, not significant; $^{*}P<0.05$ and $^{**}P<0.01$. Source data are provided in the Source Data file.

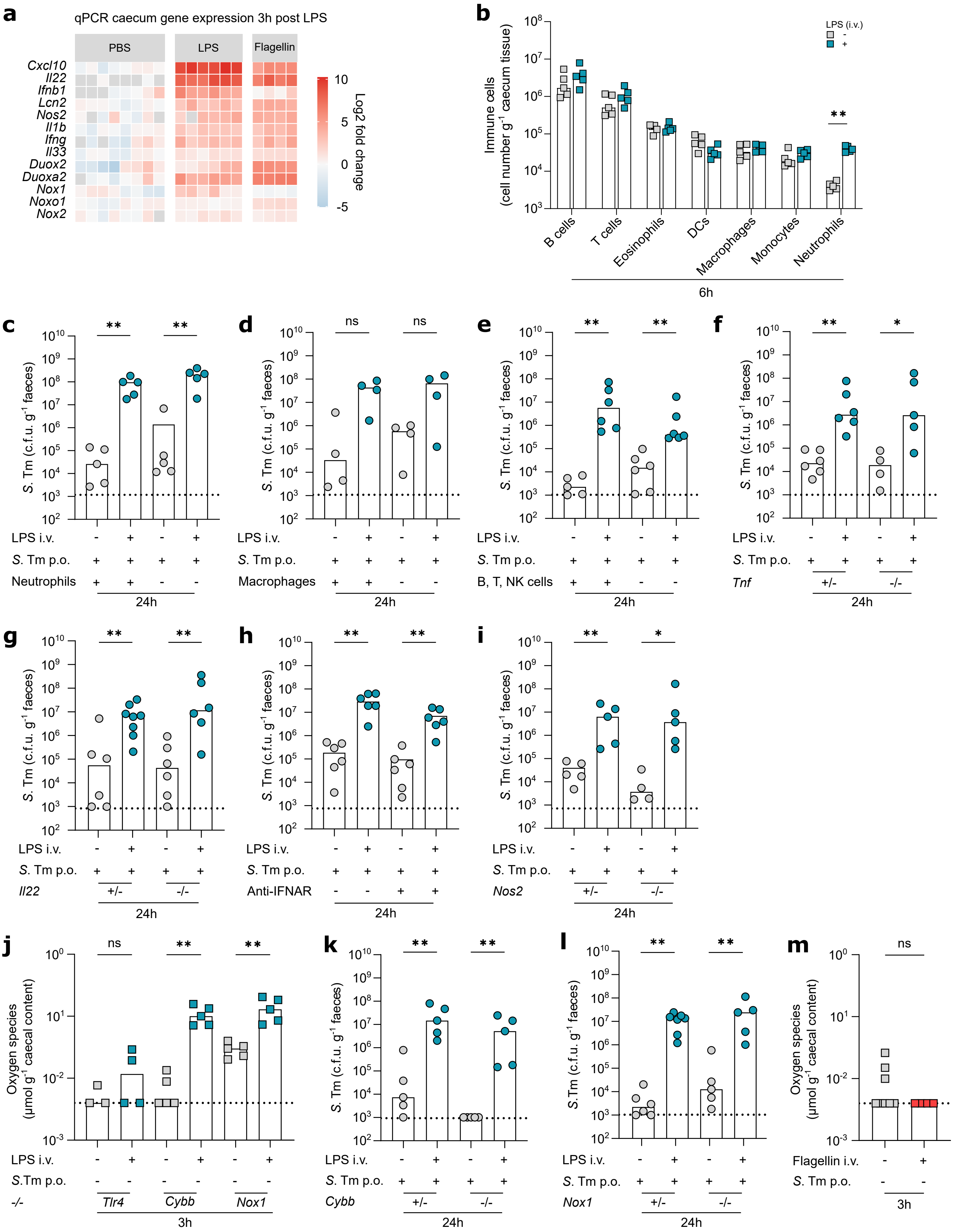

Supplementary Figure 2 Contribution of immune mediators to LPS-induced gut-luminal *S.* Tm bloom. (a) qPCR log2 fold change as determined of classical inflammatory response genes in mice systemically exposed to PBS or LPS at 3 h.p.i (minimum mice n=4, at least two independent replicates). (b) Caecum tissue immune cell composition at 6 h.p.i. in mice systemically exposed to PBS or LPS (mice

n=5, at least two independent replicates). (c) Faecal *S*. Tm loads at 24 h.p.i. in mice treated with PBS or anti-Ly6G and systemically exposed to PBS or LPS (mice n=5, at least two independent replicates). (d) Faecal *S*. Tm loads at 24 h.p.i. in mice treated with PBS or anti-CSF1R and systemically exposed to PBS or LPS (mice n=4, at least two independent replicates). (e) Faecal S. Tm loads at 24 h.p.i. in *Rag2*$^{+/-}$ *Il2rg*$^{+/-}$ and *Rag2*$^{-/-}$ *Il2rg*$^{-/-}$ littermates systemically exposed to PBS or LPS (minimum mice n=5, at least two independent replicates). (f) Faecal S. Tm loads at 24 h.p.i. in *Tnf*$^{+/-}$ and *Tnf*$^{-/-}$ littermates systemically exposed to PBS or LPS (minimum mice n=4, at least two independent replicates). (g) Faecal *S*. Tm loads at 24 h.p.i. in *Il22*$^{+/-}$ and *Il22*$^{-/-}$ littermates systemically exposed to PBS or LPS (minimum mice n=6, at least two independent replicates). (h) Faecal ^{S}. Tm loads at 24 h.p.i. in mice treated with PBS or anti-IFNAR and systemically exposed to PBS or LPS (minimum mice n=6, at least two independent replicates). (i) Faecal ^{S}. Tm loads at 24 h.p.i. in *Nos2*$^{+/-}$ and *Nos2*$^{-/-}$ littermates systemically exposed to PBS or LPS (minimum mice n=4, at least two independent replicates). (j) Caecal oxygen species levels at 3 h.p.inj. in *Tlr4*$^{-/-}$, *Cybb*$^{-/-}$, and *Nox1*$^{-/-}$ mice systemically exposed to PBS or LPS (minimum mice n=3, at least two independent replicates). (k) Faecal *S*. Tm loads at 24 h.p.i. in *Cybb*$^{+/-}$ and *Cybb*$^{-/-}$ littermates systemically exposed to PBS or LPS (minimum mice n=5, at least two independent replicates). (l) Faecal *S*. Tm loads at 24 h.p.i. in *Nox1*$^{+/-}$ and *Nox1*$^{-/-}$ littermates systemically exposed to PBS or LPS (minimum mice n=5, at least two independent replicates). (m) Caecal oxygen species levels at 3 h.p.inj. in mice systemically exposed to PBS or flagellin (minimum mice n=4, at least two independent replicates). Bars indicate median values. Dotted lines indicate conservative average limit of detection. *P* values were calculated using the two-sided Mann-Whitney U test (b-m). ns, not significant; $*P<0.05$ and $**P<0.01$. Source data are provided in the Source Data file.

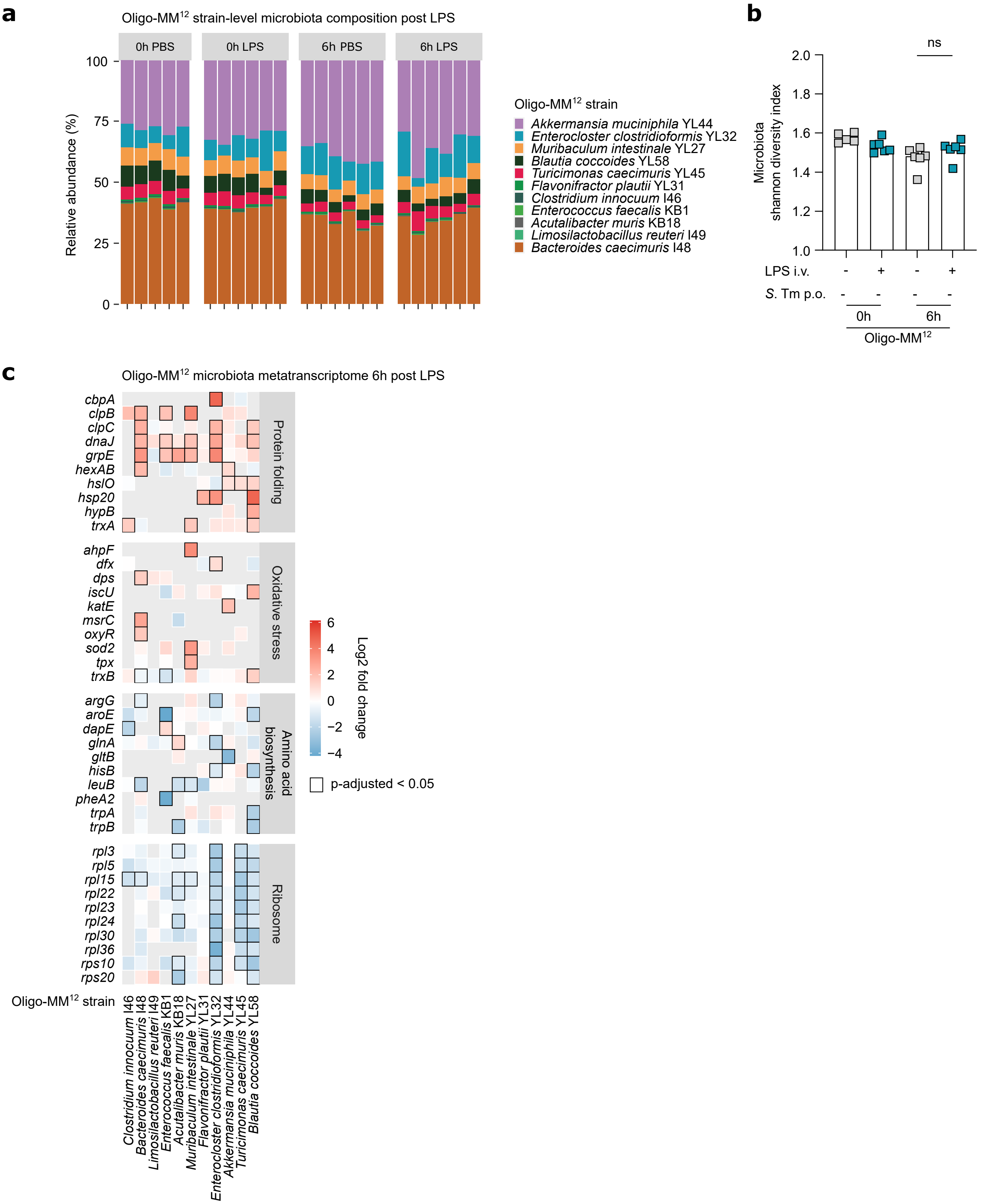

Supplementary Figure 3 Effect of systemic LPS exposure on the gut microbiota. (a) 16S rRNA sequencing strain-level microbiota composition at 0 and 6 h.p.inj. in Oligo-MM12 mice systemically exposed to PBS or LPS (minimum mice n=5, at least two independent replicates). (b) 16S rRNA sequencing Shannon diversity index at 0 and 6 h.p.inj. in Oligo-MM12 mice systemically exposed to PBS or LPS (minimum mice n=5, at least two independent replicates). (c) Caecal microbiota metatranscriptome log2 fold change of top 10 significantly differentially expressed genes involved in protein folding, oxidative stress, amino acid biosynthesis and ribosomes at 6 h.p.inj. in Oligo-MM12 mice systemically exposed to

LPS compared to PBS, (mice n=6, at least two independent replicates). Bars indicate median values. *P* values were calculated using the two-sided Mann-Whitney U test (b) or two-sided Wald test with Benjamini-Hochberg multiple test correction (c). ns, not significant. Source data are provided in the Source Data file.

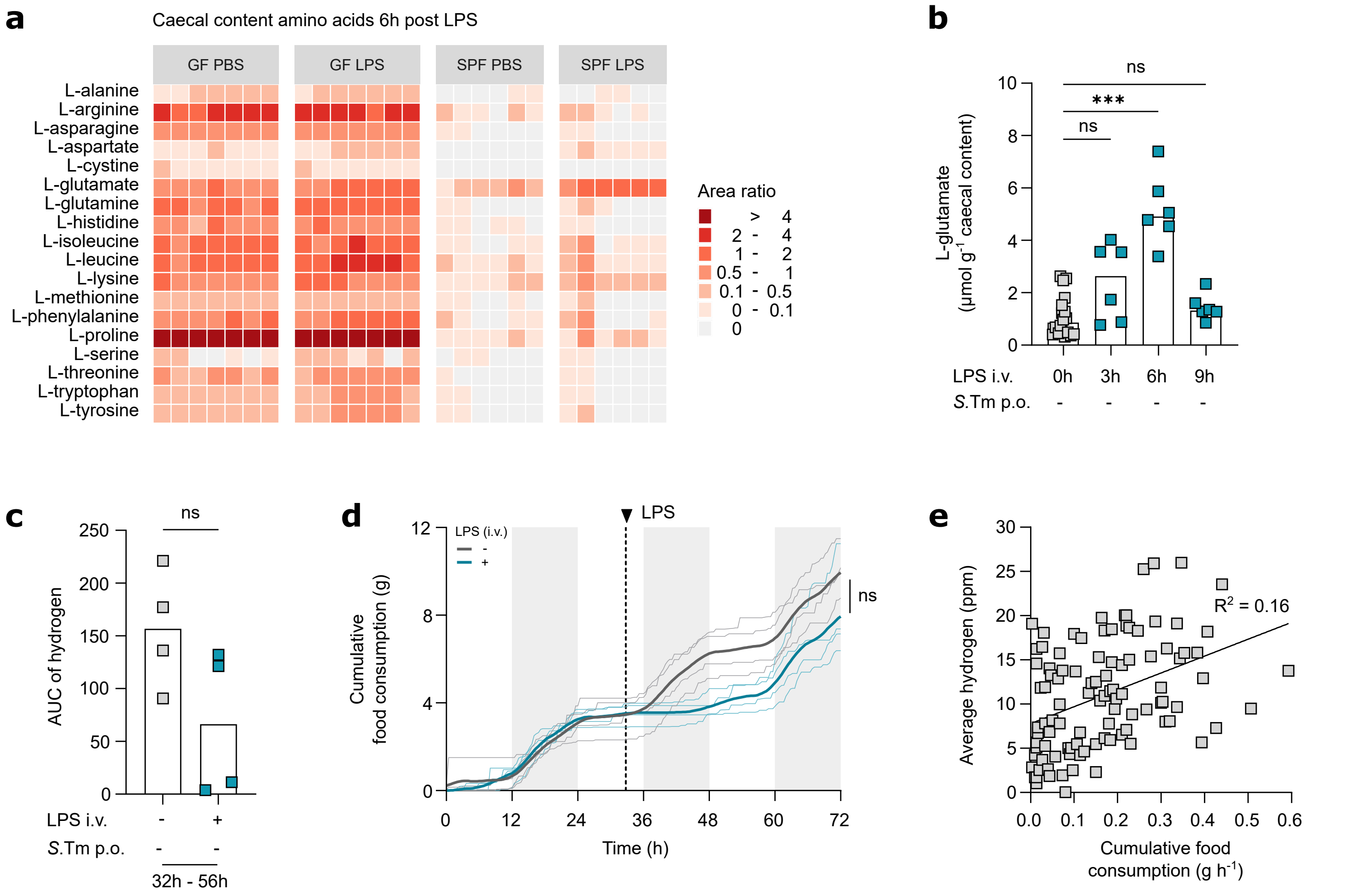

Supplementary Figure 4 Effect of systemic LPS exposure on the gut microbiota. (a) Caecal amino acid levels at 6 h.p.inj. in germ-free and SPF mice systemically exposed to PBS or LPS. Area ratio calculated to [^{13}C]-L-glutamic acid internal standard (minimum mice n=6, at least two independent replicates). (b) Caecal L-glutamate levels at 0, 3, 6 or 9 h.p.inj. in mice systemically exposed to LPS (minimum mice n=6, at least two independent replicates). (c) Area under the curve of hydrogen between 32 h and 56 h in mice systemically exposed to PBS or LPS at 32 h (minimum mice n=4, at least two independent replicates). (d) Cumulative food consumption of mice systemically exposed to PBS or LPS at 32 h, curves obtained by smoothing function of data obtained every 24 min per mouse (mice n=4, at least two independent replicates). (e) Correlation between cumulative food consumption and average hydrogen at food consumption levels calculated based on 3 h intervals. R^2 was calculated by linear regression (mice n=4, at least two independent replicates). Bars indicate median values. Dashed lines indicate time of injection. Grey rectangles indicate dark phase. *P* values were calculated using the two-sided Kruskal-Wallis test with Dunn's multiple comparisons adjustment (b) or two-sided Mann-Whitney U test (c,d). ns, not significant; ***P<0.001. Source data are provided in the Source Data file.

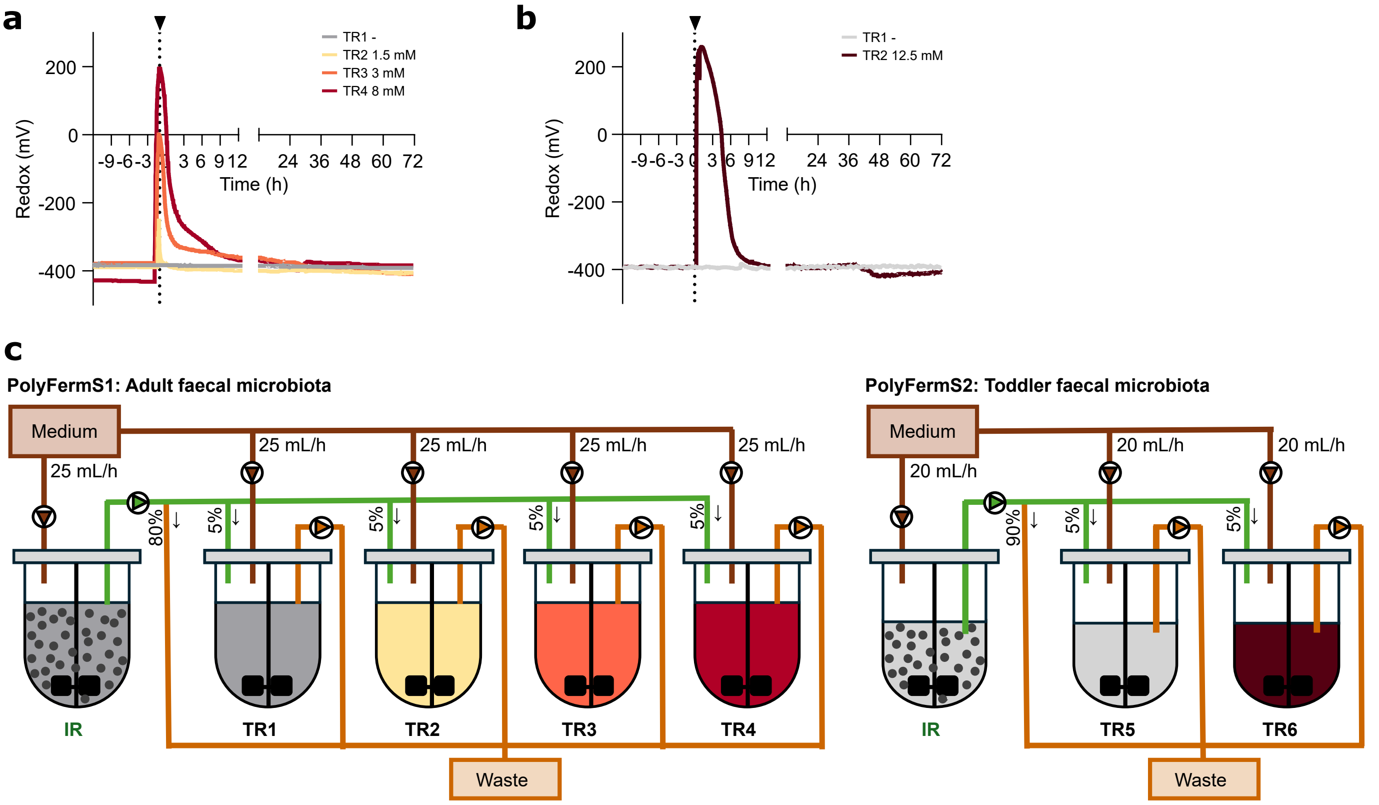

Time	Fermentation phase		PolyFerm1 - Adult TR1	TR2	TR3	TR4	PolyFerm2 - Toddler TR5	TR6
Day 1-3	Inoculation		Continuous inoculation of the TRs with 5% of the effluent from the IR					
Day 4-8	Stabilisation		Disconnection from the IR and continuous operation until metabolic stability in TRs					
Day 9-10	Treatment	Final H_2O_2 conc.:	0.0 mM	1.5 mM	3.0 mM	8.0 mM	0.0 mM	12.5 mM
		Redox potential:	-400 mV	-300 mM	0 mM	+200 mV	-400 mV	+270 mV
		∆ Redox potential:	+0 mV	+100 mV	+400 mM	+ 600 mV	+0 mV	+ 670 mV

Supplementary Figure 5 PolyFermS set-up. (a,b) Redox potential in test reactors (TR) post-H_2O_2 exposure (reactors n=4, n=2). (c) Two independent PolyFermS experiments were conducted under the proximal colon conditions of a healthy adult and a healthy toddler. Each experimental set-up included an inoculum reactor (IR), containing immobilised human faecal microbiota gel beads and consecutive TRs, which were continuously inoculated with 5% of the fermented effluent. The IR of PolyFermS1 contained immobilised faecal microbiota of an adult donor and inoculated TR1-4 while the IR of PolyFermS2 harboured immobilised faecal microbiota from a toddler and inoculated TR5 and TR6. After a 3-day-period of continuous fermentation, the TRs were disconnected from the IR and further stabilised for another 4 days to reach metabolic stability before treatment initiation. TRs were inoculated with *S*. Tm at -12h to reach a density of $1x10^8$ c.f.u. ml-1. 12h post *S*. Tm inoculation, TRs were exposed to varying H_2O_2 concentrations 0 mM (TR1,TR5), 1.5 mM (TR2), 3 mM (TR3), 8mM (TR4) and 12.5 mM (TR6) to increase the redox potential, a common measurement of the degree of anaerobiosis (reactors n=6). Dashed lines indicate time of H_2O_2 exposure. Source data are provided in the Source Data file.

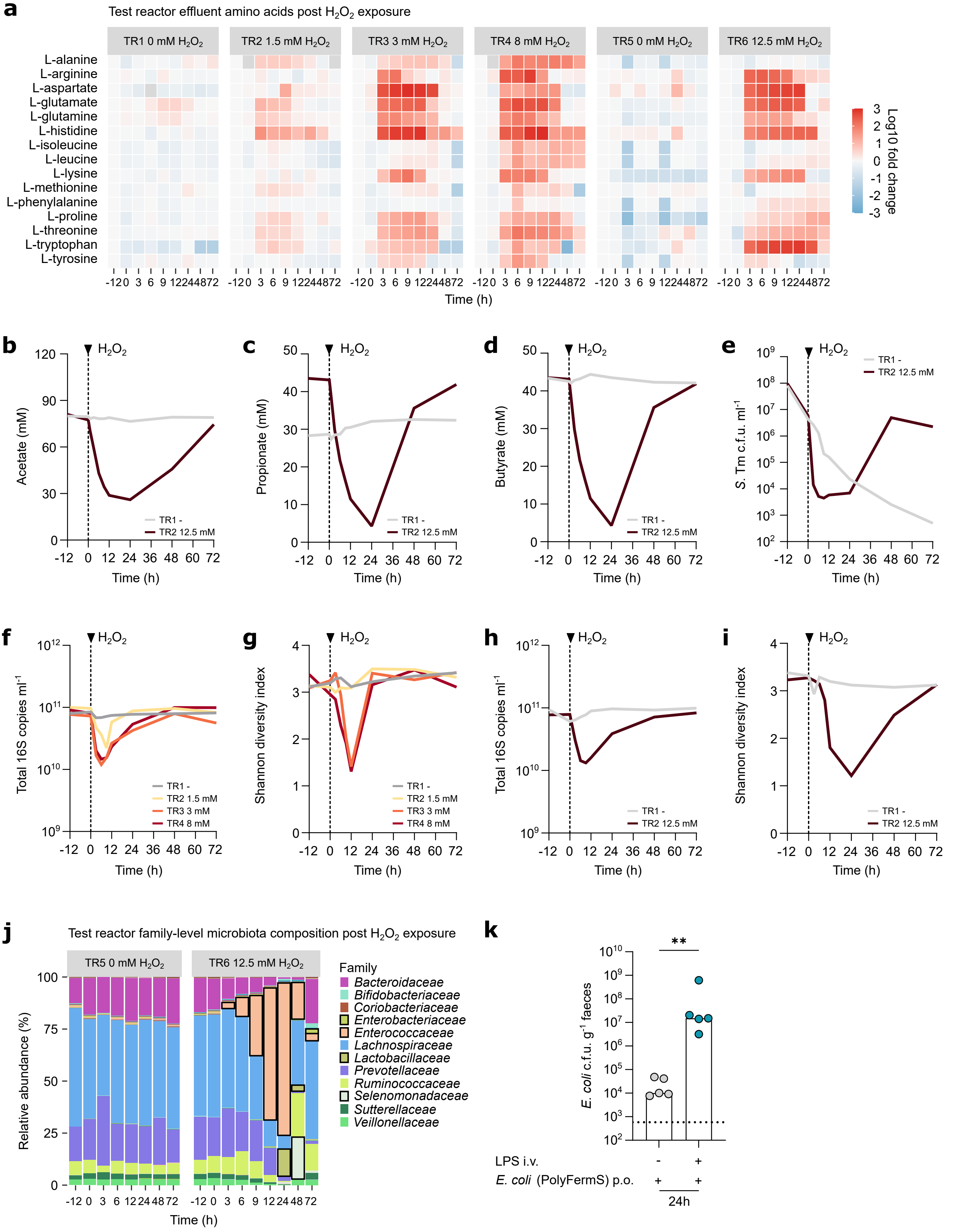

Supplementary Figure 6 Oxidative stress inhibits human gut microbiota fermentation and promotes facultative anaerobic pathogens blooms. (a) An inoculation reactor (IR) containing immobilised faecal microbiota of an adult donor inoculated TR1-4, and an IR harbouring immobilised faecal microbiota from a toddler inoculated TR5 and TR6. TRs were inoculated with S. Tm at -12h to reach a

density of 1x108 c.f.u. ml-1. 12h post *S*. Tm inoculation, TRs were exposed to varying H_2O_2 concentrations 0 mM (TR1/5), 1.5 mM (TR2), 3 mM (TR3), 8mM (TR4) and 12.5 mM (TR6) (reactors n=6). Log10 fold change amino acids in the effluent post-H_2O_2 exposure compared to respective mock TR (TR1/TR5) (reactors n=6). (b-d) Acetate, propionate, and butyrate concentrations in TR effluent post-H_2O_2 exposure (reactors n=2). (e) *S*. Tm loads in TR effluent post-H_2O_2 exposure (reactors n=2). (f-i) 16S rRNA sequencing copy number and Shannon diversity index in TR effluent post-H_2O_2 exposure (reactors n=6). (j) 16S rRNA sequencing family-level microbiota composition in TR effluent post-H_2O_2 exposure (reactors n=2). (k) Faecal *E. coli* (PolyFermS) loads at 24 h.p.i. in mice systemically exposed to PBS or LPS (mice n=5, at least two independent replicates). Dashed lines indicate time of H_2O_2 exposure. Bars indicate median values. Dotted lines indicate conservative average limit of detection. *P* values were calculated using the two-sided Mann-Whitney U test (k). ***P*<0.01. Source data are provided in the Source Data file.

a

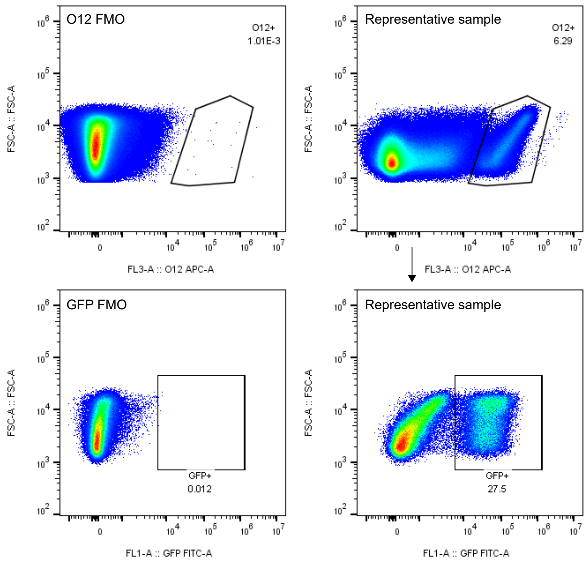

b

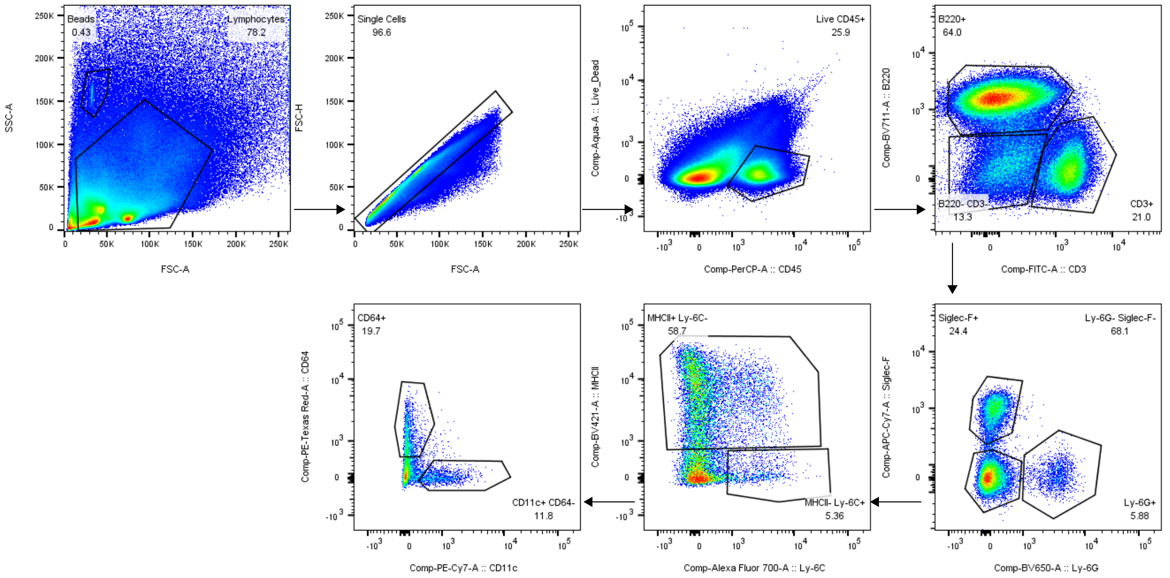

Supplementary Figure 7 Gating strategies. (a) Gating strategy for *S*. Tm *PsicA-gfp* using primary antibody human anti-*S*. Tm O12 and secondary antibody goat anti-human AF647. Including a control where the primary antibody was not added and a control *S*. Tm strain that does not express GFP. (b) Lamina propria gating strategy, initial gate was set based on size and granularity, live CD45$^+$ cells, CD3$^+$ T cells, B220$^+$ B cells, Ly-6G$^+$ neutrophils, Siglec-F$^+$ eosinophils, Ly-6G$^-$ Ly-6C$^+$ monocytes, CD64$^+$ macrophages and CD11c$^+$ dendritic cells.

Supplementary Table 1 Bacterial strains

Relevant genotype	Strain number	Reference
Wild type *S.* Tm SL1344	SB300	Hoiseth & Stocker 1981[88]
SB300 Δ*sopB* Δ*sipA* Δ*sopE* Δ*sopE2*	M566	Ehrbar 2003[121]
SB300 Δ*cyxA*:cat *WITS17*:aphT	Z6860	This study
SB300 Δ*cydB*:cat *WITS19*:aphT	Z6889	This study
SB300 Δ*cyoA*:cat *WITS17*:ahpT	T1728	This study
SB300 Δ*cyxA*:cat Δ*cydB WITS19*:ahpT	Z6859	This study
SB300 Δ*gltA*:cat *WISH1*:bla	Z8390	Nguyen 2024[37]
SB300 Δ*acnA*:aphT Δ*acnB*:cat *WISH3*:bla	Z8382	Nguyen 2024[37]
SB300 Δ*icdA*:aphT *WISH4*:bla	Z8384	Nguyen 2024[37]
SB300 Δ*sucB*:aphT *WISH5*:bla	Z8386	Nguyen 2024[37]
SB300 Δ*sucD*:cat *WITS2*:aphT	T1703	This study
SB300 Δ*sdh*:cat *WITS13*:aphT	Z3436	Nguyen 2020[68]
SB300 Δ*dcuA* Δ*dcuB* Δ*dcuC* Δ*frd WITS2*:aphT	Z3444	Nguyen 2020[68]
SB300 Δ*fumAC* Δ*fumB WITS17*:aphT	Z3460	Nguyen 2020[68]
SB300 Δ*mdh*:cat *WITS19*:aphT	Z3468	This study
E. coli	CFT073	Mobley 1990[122]
K. pneumoniae	T821	This study
E. faecium	T1749	This study
E. coli (PolyFermS)	T1740	This study
Agathobacter rectalis	A1-86	DSM No.: 17629
Bacteroides fragilis	EN-2	DSM No.: 2151
Bacteroides thetaiotaomicron	EP50	DSM No.: 2079
Bacteroides uniformis	VPI 0061	DSM No.: 6597
Clostridium perfringens	C36	DSM No.: 11782
Collinsella aerofaciens	VPI 1003	DSM No.: 3979
Coprococcus comes	VPI CI-38	ATCC No.: 27758
Dorea formicigenerans	VPI C8-13	DSM No.: 3992
Enterocloster bolteae	WAL 16351	DSM No.: 15670
Escherichia coli	BW25113	PMID:10829079
Fusobacterium nucleatum	VPI 4355	DSM No.: 15643
Lacrimispora saccharolytica	WM1	DSM No.: 2544
Parabacteroides merdae	VPI T4-1	DSM No.: 19495
Phocaeicola vulgatus	LRA 049 07 85	DSM No.: 1447
Roseburia intestinalis	L1-82	DSM No.: 14610
Ruminococcus gnavus	VPI C7-9	ATCC No.: 29149
Streptococcus parasanguinis	SS 898	DSM No.: 6778
Streptococcus salivarius	275	DSM No.: 20219
Thomasclavelia ramosa	113-I	DSM No.: 1402
Bacteroides caecimuris	I48	Brugiroux 2016[55]
Bifidobacterium animalis	YL2	Brugiroux 2016[55]
Blautia pseudococcoides	YL58	Brugiroux 2016[55]
Clostridium innocuum	I46	Brugiroux 2016[55]
Enterocloster clostridioformis	YL32	Brugiroux 2016[55]
Enterococcus faecalis	KB1	Brugiroux 2016[55]
Flavonifractor plautii	YL31	Brugiroux 2016[55]
Limosilactobacillus reuteri	I49	Brugiroux 2016[55]

Supplementary Table 2 RT^2 qPCR primers

Gene	Species	Ref.Seq.	Cat.Nr.
Actb	Mouse	NM_007393	PPM02945B-200
Cxcl10	Mouse	NM_021274	PPM02978E-200
Il22	Mouse	NM_016971	PPM05481A-200
Ifnb1	Mouse	NM_010510	PPM03594C-200
Lcn2	Mouse	NM_008491.1	PPM03770A-200
Nos2	Mouse	NM_010927	PPM02928B-200
Il1b	Mouse	NM_008361	PPM03109F-200
Ifng	Mouse	NM_008337	PPM03121A-200
Il33	Mouse	NM_133775	PPM32527A-200
Duox2	Mouse	NM_177610	PPM40846A-200
Duoxa2	Mouse	NM_025777	PPM32328A-200
Nox1	Mouse	NM_172203	PPM34199A-200
Noxo1	Mouse	NM_027988	PPM36220A-200
Cybb	Mouse	NM_007807	PPM32951A-200

Supplementary Table 3 Antibodies

Antigen	Fluorophore	Company	Clone	Cat #	Lot #	Dilution
Fixable live/dead dye	Aqua	Thermo Fisher Scientific		L34957	2335578	1:1000
CD45	PerCP	Biolegend	30-F11	103130	B236192	1:100
CD3	FITC	Biolegend	17A2	100203	B388790	1:100
B220	BV711	Biolegend	RA3-6B2	103255	B305860	1:200
Ly-6G	BV650	Biolegend	1A8	127641	B314454	1:100
Siglec-F	APC-Cy7	BD Bioscience	E50-2440	565527	1062707	1:200
MHCII	BV421	Biolegend	M5/114.15.2	107632	B335578	1:100
Ly-6C	AF700	Biolegend	HK1.4	128024	B318988	1:200
CD64	PE/Dazzle	Biolegend	X54-5/7.1	139320	B304964	1:100
CD11c	PE-Cy7	Biolegend	N418	117318	B264758	1:200

References

121. Ehrbar, K., Friebel, A., Miller, S. I. & Hardt, W. D. Role of the Salmonella Pathogenicity Island 1 (SPI-1) Protein InvB in Type III Secretion of SopE and SopE2, Two Salmonella Effector Proteins Encoded Outside of SPI-1. *J. Bacteriol.* **185**, 6950–6967 (2003). doi:10.1128/JB.185.23.6950-6967.2003.

122. Mobley, H. L. *et al.* Pyelonephritogenic Escherichia coli and killing of cultured human renal proximal tubular epithelial cells: Role of hemolysin in some strains. *Infect. Immun.* **58**, 1281–1289 (1990). doi:10.1128/iai.58.5.1281-1289.1990.
