# Supplementary material for: Sublethal systemic LPS in mice enables gut-luminal pathogens to bloom through oxygen species-mediated microbiota inhibition
Source: Nat Commun. 2025 Mar 20;16:2760. doi: 10.1038/s41467-025-57979-0 (PMC11926250; doi:10.1038/s41467-025-57979-0)
Supplement: Supplementary file 3 — Reporting Summary [file 41467_2025_57979_MOESM3_ESM.pdf]

nature portfolio

Corresponding author(s): Annika Hausmann and Wolf-Dietrich Hardt

Last updated by author(s): Jan 17, 2025

Reporting Summary

Nature Portfolio wishes to improve the reproducibility of the work that we publish. This form provides structure for consistency and transparency in reporting. For further information on Nature Portfolio policies, see our Editorial Policies and the Editorial Policy Checklist.

Statistics

For all statistical analyses, confirm that the following items are present in the figure legend, table legend, main text, or Methods section.

n/a	Confirmed	
☐	☒	The exact sample size (*n*) for each experimental group/condition, given as a discrete number and unit of measurement
☐	☒	A statement on whether measurements were taken from distinct samples or whether the same sample was measured repeatedly
☐	☒	The statistical test(s) used AND whether they are one- or two-sided<br>*Only common tests should be described solely by name; describe more complex techniques in the Methods section.*
☒	☐	A description of all covariates tested
☒	☐	A description of any assumptions or corrections, such as tests of normality and adjustment for multiple comparisons
☐	☒	A full description of the statistical parameters including central tendency (e.g. means) or other basic estimates (e.g. regression coefficient) AND variation (e.g. standard deviation) or associated estimates of uncertainty (e.g. confidence intervals)
☐	☒	For null hypothesis testing, the test statistic (e.g. *F*, *t*, *r*) with confidence intervals, effect sizes, degrees of freedom and *P* value noted<br>*Give P values as exact values whenever suitable.*
☒	☐	For Bayesian analysis, information on the choice of priors and Markov chain Monte Carlo settings
☒	☐	For hierarchical and complex designs, identification of the appropriate level for tests and full reporting of outcomes
☒	☐	Estimates of effect sizes (e.g. Cohen's *d*, Pearson's *r*), indicating how they were calculated

Our web collection on statistics for biologists contains articles on many of the points above.

Software and code

Policy information about availability of computer code

Data collection	Data was collected as described in the manuscript.
Data analysis	All data was analysed using open source tools or tools that have been used under appropriate academic licenses. All data was plotted using GraphPad Prism 9 or using ggplot2 in R v4.3.2 with R Studio v2023.09.1. The specific tools and packages used are detailed below:<br><br>Caecum Tissue Analysis: mRNA reads were mapped to the mouse genome using STAR. Reads were quantified using featureCounts. Differential expression analysis was performed using edgeR. Over-representation analysis was carried out with WebGestalt 2019 (accessible at https://www.webgestalt.org/).<br><br>16S rRNA Analysis: Reads were trimmed using cutadapt and analysed with the dada2 pipeline. ASVs were taxonomically annotated using IDTAXA in combination with the Silva v138 database (https://www.arb-silva.de/).<br><br>Microbiota mRNA Analysis: Reads were cleaned by removing adaptor sequences, performing low-quality-end trimming, and excluding low-quality reads using BBTools v38.18 (https://sourceforge.net/projects/bbmap/). The exact commands and parameters for quality control are provided in the Methods in Microbiomics documentation (https://methods-in-microbiomics.readthedocs.io/en/latest/preprocessing/preprocessing.html). Reads were mapped to concatenated genomes of Oligo-MM12 bacterial strains using bowtie2 and quantified with featureCounts. Differential expression analysis was conducted using DESeq2 with taxon-specific normalization.<br><br>Flow Cytometry Data Analysis: Data was analysed using FlowJo 10.

Single-Strain Bacterial Growth Curve Analysis: Growth curve data was analysed using the R package neckaR (https://github.com/Lisa-Maier-Lab/neckaR).

For manuscripts utilizing custom algorithms or software that are central to the research but not yet described in published literature, software must be made available to editors and reviewers. We strongly encourage code deposition in a community repository (e.g. GitHub). See the Nature Portfolio guidelines for submitting code & software for further information.

Data

Policy information about availability of data

All manuscripts must include a data availability statement. This statement should provide the following information, where applicable:

- Accession codes, unique identifiers, or web links for publicly available datasets
- A description of any restrictions on data availability
- For clinical datasets or third party data, please ensure that the statement adheres to our policy

The data generated in this study are provided as Source Data files. The raw sequencing data generated in this study have been deposited in the European Nucleotide Archive (ENA) under accession number PRJEB82444 (https://www.ebi.ac.uk/ena/browser/view/PRJEB82444) and in the NCBI BioSample database under accession number SAMN45075194 (https://www.ncbi.nlm.nih.gov/biosample/?term=SAMN45075194).

Research involving human participants, their data, or biological material

Policy information about studies with human participants or human data. See also policy information about sex, gender (identity/presentation), and sexual orientation and race, ethnicity and racism.

Reporting on sex and gender	N/A
Reporting on race, ethnicity, or other socially relevant groupings	N/A
Population characteristics	N/A
Recruitment	N/A
Ethics oversight	N/A

Note that full information on the approval of the study protocol must also be provided in the manuscript.

Field-specific reporting

Please select the one below that is the best fit for your research. If you are not sure, read the appropriate sections before making your selection.

[x] Life sciences [ ] Behavioural & social sciences [ ] Ecological, evolutionary & environmental sciences

For a reference copy of the document with all sections, see nature.com/documents/nr-reporting-summary-flat.pdf

Life sciences study design

All studies must disclose on these points even when the disclosure is negative.

Sample size	A minimum of five mice per group was used in this study. Sample sizes were chosen based on institutional guidelines and in adherence to the 3Rs principles (Replacement, Reduction, and Refinement) to uphold ethical standards in animal research. No statistical method was used to predetermine sample size. A minimum of five mice per group was deemed sufficient to observe consistent and reproducible results across experimental groups, based on prior studies with similar experimental designs.
Data exclusions	No data were excluded from the analysis.
Replication	All the experiments were performed at least twice, with all attempts at data replication being successful.
Randomization	Mice were randomly assigned to experimental groups to ensure generalisability of results.
Blinding	Blinding was not applicable, as investigators needed to identify the cages of mice for subsequent treatments or infections with respective bacterial strains.

Reporting for specific materials, systems and methods

We require information from authors about some types of materials, experimental systems and methods used in many studies. Here, indicate whether each material, system or method listed is relevant to your study. If you are not sure if a list item applies to your research, read the appropriate section before selecting a response.

Materials & experimental systems

n/a	Involved in the study
☐	☒ Antibodies
☒	☐ Eukaryotic cell lines
☒	☐ Palaeontology and archaeology
☐	☒ Animals and other organisms
☒	☐ Clinical data
☒	☐ Dual use research of concern
☒	☐ Plants

Methods

n/a	Involved in the study
☒	☐ ChIP-seq
☐	☒ Flow cytometry
☒	☐ MRI-based neuroimaging

Antibodies

Antibodies used

CD45 PerCP Biolegend Clone 30-F11 Cat # 103130 Lot # B236192 Dilution 1:100
CD3 FITC Biolegend Clone 17A2 Cat # 100203 Lot # B388790 Dilution 1:100
B220 BV711 Biolegend Clone RA3-6B2 Cat #103255 Lot # B305860 Dilution 1:200
Ly-6G BV650 Biolegend Clone 1A8 Cat #127641 Lot # B314454 Dilution 1:100
Siglec-F APC-Cy7 Clone BD Bioscience E50-2440 Cat #565527 Lot # 1062707 Dilution 1:200
MHCII BV421 Biolegend Clone M5/114.15.2 Cat 107632 Lot # B335578 Dilution 1:100
Ly-6C AF700 Biolegend Clone HK1.4 Cat #128024 Lot # B318988 Dilution 1:200
CD64 PE/Dazzle Biolegend Clone X54-5/7.1 Cat #139320 Lot # B304964 Dilution 1:100
CD11c PE-Cy7 Biolegend Clone N418 Cat #117318 Lot # B264758 Dilution 1:200
human anti-S. Tm O12 antibody (hSTA5) kind gift of Antonio Lanzavecchia, Institute for Research in Biomedicine, Bellinzona, Switzerland Dilution 1:200
Goat anti-human IgG Fcγ fragment specific AF647 Jackson ImmunoResearch Europe Cat #109-605-098 Dilution 1:200

Validation

The antibodies were validated by the manufacturer:
CD45 https://www.biolegend.com/en-gb/products/percp-anti-mouse-cd45-antibody-4265
CD3 https://www.biolegend.com/en-us/products/fitc-anti-mouse-cd3-antibody-45
B220 https://www.biolegend.com/en-us/products/brilliant-violet-711-anti-mouse-human-cd45r-b220-antibody-9692
Ly-6G https://www.biolegend.com/en-us/products/brilliant-violet-650-anti-mouse-ly-6g-antibody-11981
Siglec-F https://www.bdbiosciences.com/en-us/products/reagents/flow-cytometry-reagents/research-reagents/single-color-antibodies-ruo/apc-cy-7-rat-anti-mouse-siglec-f.565527
MHCII https://www.biolegend.com/en-us/products/brilliant-violet-421-anti-mouse-i-a-i-e-antibody-7147
Ly-6C https://www.biolegend.com/en-us/products/alexa-fluor-700-anti-mouse-ly-6c-antibody-6757
CD64 https://www.biolegend.com/en-us/products/pe-dazzle-594-anti-mouse-cd64-fcgammari-antibody-12424
CD11c https://www.biolegend.com/en-us/products/pe-cyanine7-anti-mouse-cd11c-antibody-3086
For bacterial flow cytometry (human anti-S. Tm O12 antibody (hSTA5) + Goat anti-human IgG Fcγ fragment specific AF647), fluorescence minus one was used to determine cutoffs as shown in Supplementary Figure 7a

Animals and other research organisms

Policy information about studies involving animals; ARRIVE guidelines recommended for reporting animal research, and Sex and Gender in Research

Laboratory animals

8- to 12-week old mice were held under specific pathogen-free (SPF) conditions at the ETH Phenomics Centre (EPIC) at ETH Zürich (light/dark cycle 12:12 h, room temperature 21±1 ˚C, humidity 50±10%). Germ-free and Oligo-MM12 mice were bred in flexible film isolators at the isolator facility at the EPIC facility.
The following mouse lines were used:
C57BL/6J (WT; Ly5.2, in-house breeding, JAX:000664, The Jackson Laboratory)
Tlr4-/- (B6.129-Tlr4tm1Aki/Aki) Hoshino, K. (1999) https://doi.org/10.4049/jimmunol.162.7.3749
Casp11-/- (B6.B6-Casp11tm1) Kayagaki, N (2011) https://doi.org/10.1038/nature10558
Rag2-/- Il2rg-/- (B6.B6(Rag2tm1Fwa)(Il2rgtm1Cgn)) Shinkai, Y. (1992) https://doi.org/10.1016/0092-8674(92)90029-C, Disanto, J. P. (1995) https://doi.org/10.1073/pnas.92.2.377
Tnf-/- (B6.129-Tnfatm1Ljo) Marino, M. W. (1997) https://doi.org/10.1073/pnas.94.15.8093
Il22-/- (B6.129S5-Il22tm1Lex) Zheng, Y. (2007) https://doi.org/10.1038/nature05505
Nos2-/- (B6.129P2-Nos2tm1Lau/J) Laubach, V. E. (1995) https://doi.org/10.1073/pnas.92.23.10688
Cybb-/- (B6.129S-Cybbtm1Din/J) Pollock, J. D. (1995) https://doi.org/10.1038/ng0295-202
Nox1-/- (B6.129X1-Nox1tm1Kkr/J) Gavazzi, G. (2006) https://doi.org/10.1016/j.febslet.2005.12.049
All the mice used had a C57BL/6J background

Wild animals

No wild animals were used in this study.

Reporting on sex

Mice of both sexes were randomly assigned to experimental groups to ensure generalisability of results.

Field-collected samples

This study did not involve samples collected from the field.

Ethics oversight	All animal experiments were reviewed and approved by Kantonales Veterinäramt Zürich under license ZH158/2019 and ZH108/2022, complying with the cantonal and Swiss legislation.

Note that full information on the approval of the study protocol must also be provided in the manuscript.

Plants

Seed stocks	N/A
Novel plant genotypes	N/A
Authentication	N/A

Flow Cytometry

Plots

Confirm that:

- [x] The axis labels state the marker and fluorochrome used (e.g. CD4-FITC).
- [x] The axis scales are clearly visible. Include numbers along axes only for bottom left plot of group (a 'group' is an analysis of identical markers).
- [x] All plots are contour plots with outliers or pseudocolor plots.
- [x] A numerical value for number of cells or percentage (with statistics) is provided.

Methodology

Sample preparation	For caecum lamina propria cell isolation, epithelial cells were dislodged in PBS supplemented with 5 mM EDTA (Thermo Fisher Scientific), 15 mM HEPES (Thermo Fisher Scientific) and 10% heat-inactivated FCS (Thermo Fisher Scientific). Tissue was digested into a single-cell suspension in RPMI (Thermo Fisher Scientific) supplemented with 1 mg collagenase VIII (Sigma-Aldrich) and 0.2 mg DNAse (Roche). Cells were incubated in 1 µg per sample Mouse BD Fc Block (BD Biosciences) in 10% Brilliant stain buffer (BD Biosciences)/FACS buffer for 5 min at 4°C. Followed by staining with the antibody mix for 30 min at 4°C (Extended Data Table 3). Samples were measured on a LSR Fortessa (BD Biosciences), and data was analysed with FlowJo V10 (TreeStar).<br><br>Activity of the sicA promoter was analysed in mice using a plasmid-based PsicA-gfp reporter. Fresh caecum content was collected 24 h.p.i. and diluted in PBS. Caecum content was incubated for 1 h at room temperature with 2 µg ml-1 of chloramphenicol (AppliChem) to inhibit protein synthesis and allow GFP proteins to fully mature. S. Tm was stained with a human anti-S. Tm O12 antibody (hSTA5; kind gift of Antonio Lanzavecchia, Institute for Research in Biomedicine, Bellinzona, Switzerland), and goat anti-human IgG AF647 antibody (Jackson ImmunoResearch Europe). Fluorescence was measured with a Cytoflex flow cytometer (Beckman Coulter) acquired with CytExpert software v.2.5.
Instrument	LSR Fortessa (BD Biosciences), Beckman Coulter CytoflexS
Software	DIVA, FlowJo V10, CytExpert software v.2.5
Cell population abundance	For lamina propria cell isolation, approximately 10% of acquired events were live CD45+ cells.<br>For S. Tm GFP analysis, approximatley 6% of acquired events stained positive for S. Tm O12
Gating strategy	Lamina propria gating strategy, initial gate was set based on size and granularity. All other gates were set based on pseudocolor cut-offs. live CD45+ cells, CD3+ T cells, B220+ B cells, Ly-6G+ neutrophils, Siglec-F+ eosinophils, Ly-6G- Ly-6C+ monocytes, CD64+ macrophages and CD11c+ DCs.<br>Gating strategy for S. Tm PsicA-gfp using primary antibody human anti-S. Tm O12 and secondary antibody goat anti-human AF647. Including a fluorescence minus one control where the primary antibody was not added and a control S. Tm strain that does not express GFP.

- [x] Tick this box to confirm that a figure exemplifying the gating strategy is provided in the Supplementary Information.
